# Effectiveness of oral dydrogesterone compared to placebo in reducing the risk of preterm birth: a systematic review and meta-analysis

**DOI:** 10.1186/s12884-026-08747-5

**Published:** 2026-02-24

**Authors:** Sohieb Hedawy, Shahed Aldalahmeh, Eman E. Labeeb, Abdelrahman A. Khattab, Esraa Khalid, Ahmed Hassan, Ahmed Menshawy

**Affiliations:** 1 Faculty of Medicine, Al-Azhar Assiut University, Assiut, Egypt; 2 Faculty of Medicine, Jordan University of Science and Technology, Al- Ramtha, Jordan; 3 Faculty of Medicine, Al Azhar University, Cairo, Egypt; 4 Faculty of Medicine, Misr University for Science and Technology, Giza, Egypt; 5 Department of Clinical Pharmacy, Faculty of Pharmacy, University of Sadat City, Sadat City, Menoufia 32897 Egypt; 6 OB/GYN Resident, Faculty of Medicine, Al Hussien University Hospital, Al Azhar University, Cairo, Egypt

**Keywords:** Dydrogesterone, Preterm birth, Preterm labor, Progesterone, Meta-analysis, Systematic review, Neonatal outcomes

## Abstract

**Background:**

Preterm birth remains a major contributor to neonatal morbidity and mortality worldwide. Oral dydrogesterone is used in some clinical settings for preterm birth prevention, despite uncertainty regarding its effectiveness. This systematic review aimed to evaluate the efficacy and safety of oral dydrogesterone compared with placebo in women at risk of preterm birth.

**Methods:**

We conducted a systematic review and meta-analysis in accordance with PRISMA guidelines. Randomized controlled trials comparing oral dydrogesterone with placebo in pregnant women at risk of preterm birth were included; non-randomized, observational, and animal studies were excluded. We searched PubMed, Cochrane Library, Web of Science, and Scopus from inception to December 2024. Risk of bias was assessed using the Cochrane Risk of Bias tool. Random-effects meta-analyses were performed where appropriate. We evaluated Certainty of evidence using the GRADE approach, and trial sequential analysis (TSA) was conducted to assess the conclusiveness of the evidence. The review was registered in PROSPERO (CRD42025639288).

**Results:**

Five randomized controlled trials involving 436 participants were included. Compared with the placebo, dydrogesterone showed a non-significant trend toward increased gestational age at delivery (mean difference, 0.42 weeks; 95% CI -0.70 to 1.55) and a reduced risk of neonatal intensive care unit admission (risk ratio, 0.74; 95% CI 0.47 to 1.18). No statistically significant differences were observed for other maternal or neonatal outcomes. The certainty of evidence was rated as low or very low for most outcomes using GRADE. TSA demonstrated that the accumulated evidence remains underpowered and insufficient to draw firm conclusions.

**Conclusion:**

Current randomized evidence does not demonstrate a statistically or clinically meaningful benefit of oral dydrogesterone over placebo in preventing preterm birth or improving maternal or neonatal outcomes. The low to very low certainty of evidence and inconclusive TSA findings indicate that the existing evidence base is insufficient to support clinical recommendations. Larger, high-quality randomized trials are required before oral dydrogesterone can be considered for routine clinical use.

**Supplementary Information:**

The online version contains supplementary material available at 10.1186/s12884-026-08747-5.

## Background

Preterm labor (PTL) is characterized as spontaneous labor occurring before 37 weeks of gestation, while the typical human pregnancy lasts approximately 40 weeks (9.2 months) [1]. Each year, approximately 15 million preterm neonates are born, accounting for one in every ten infants, with this number continuing to rise globally. This makes preterm birth a major challenge in obstetrics and perinatal care worldwide [2–5]. A population-based analysis of infant deaths indicates that PTL accounts for almost 27% of annual fatalities [4]. Although survival rates have improved due to advancements in neonatal intensive care, PTL can result in prematurity, placing infants at an elevated risk for numerous morbidities, including intraventricular hemorrhage (IVH), respiratory distress syndrome (RDS), neonatal sepsis, necrotizing enterocolitis (NEC), significant visual and neurodevelopmental impairments, and potential mortality [5]. The diagnosis of PTL is typically based on clinical criteria, including the presence of regular uterine contractions alongside alterations in cervical dilatation, effacement, or both [6].

An essential concern in healthcare is the efficacy of PTL management. Tocolytic treatment and corticosteroids are used to delay delivery and manage premature labor. Tocolysis is administered for a duration of 48 hours [7]. Tocolytic treatment is primarily utilized to control PTL; Additionally, it allows sufficient time for prenatal corticosteroid therapy to expedite fetal lung maturation. The predominant tocolytic agents that have been extensively studied include indomethacin, nifedipine, and magnesium sulfate (MgSO_4_) [7].

Numerous studies have been undertaken to concentrate on the prevention of premature labor. Progestogen compounds are among the most promising agents for the prevention of premature labor [8]. Recent data indicate that progesterone plays a crucial role in maintaining uterine quiescence throughout the latter half of pregnancy by limiting the generation of stimulatory prostaglandins and inhibiting the expression of contraction-associated protein genes. Moreover, the functional withdrawal of progesterone is believed to trigger labor [9]. Exogenous progesterone (PRG) has been studied for its preventive function in PTL and is featured in new guidelines that advocate for progesterone as a key preventive agent in singleton pregnancies with a history of spontaneous vaginal delivery or reduced cervical length [10].

Numerous studies have investigated the administration of progesterone supplements in patients experiencing PTL. While some research indicated that progesterone maintenance therapy following successful tocolysis substantially extends the latency period, other investigations have failed to support this finding [11]. In vitro studies examining various progesterones revealed that only dydrogesterone, a potent orally active progestogen with a molecular structure and pharmacological effects similar to endogenous progesterone, exhibited a rapid and direct inhibition of myometrial contraction when administered at a suitable dose and time [12].

Dydrogesterone (6-dehydro-9b, 10a-progesterone) is a synthetic progestational hormone with no estrogenic, androgenic, anti-androgenic, or glucocorticoid effects, as it binds almost exclusively to the progesterone receptor [13, 14]. It has good oral bioavailability and causes an increase in the endometrial thickness at a dose 10–20 times lower than that of micronized progesterone [15]. It has been used in many gynecological and obstetric complications, such as threatened abortion [12], regularization, and reducing menstrual pain and anxiety [16], luteal phase support [17], and in hormonal replacement therapy [18].

Despite the ambiguous supporting evidence regarding the use of dydrogesterone in the prevention of preterm delivery in individuals at risk of preterm birth, it has been widely utilized in some countries [19, 20].

In our systematic review and meta-analysis, we aim to synthesize and evaluate evidence on the safety and efficacy of using dydrogesterone to prevent the risk of preterm birth compared to placebo. Specifically, we assess the incidence of recurrence of uterine contractions, the latency period, and neonatal and pregnancy complications.

## Methods

This systematic review and meta-analysis were performed according to the Preferred Reporting Items for Systematic Reviews and Meta-Analyses (PRISMA) guidelines and in accordance with the Cochrane Handbook for Systematic Reviews of Interventions. The study was registered and published in PROSPERO with ID CRD42025639288.

### Search strategy and selection criteria

One investigator searched the PubMed, Cochrane Library, Web of Science, and Scopus databases up to 8 December 2024, and we conducted an updated search through 11 November 2025. The search strategy included the Medical Subject Headings (MeSH) and text words for the following terms: “Dydrogesterone” and “preterm delivery”. Detailed search strategies are provided in Table S1.

The inclusion criteria were randomized controlled trials published in international, peer-reviewed journals that investigated the effect of dydrogesterone compared to placebo in pregnant women at risk of preterm delivery. There were no restrictions on language, age, or country.

The exclusion criteria were as follows: (a) not a randomized clinical trial such as reviews, observation studies, case series, case reports, conference abstracts, editorial, and animal studies; (b) studies that did not align with our PICO criteria, such as those focused on miscarriage or abortion; (c) unavailable full texts; (d) retracted studies.

Duplicates were removed using EndNote software (version 21). Two independent reviewers screened the articles using an Excel spreadsheet in two phases: (1) title and abstract screening, followed by (2) full-text screening. Studies meeting the eligibility criteria were included. A third independent reviewer was consulted to resolve any conflict.

### Data extraction and quality assessment

Two independent investigators used an Excel spreadsheet to extract the following data from eligible studies: first author’s name, year of publication, country, study aim, intervention and comparison agents, number of patients in each group, primary endpoints, and conclusions.

Baseline characteristics included: maternal age, gestational age at admission, maternal body mass index (BMI), number of primigravida and nulliparous women, and history of preterm birth.

Extracted outcomes included: recurrence of uterine contractions, latency period, gestational age at delivery, mode of delivery, pregnancy complications, neonatal complications, birth weight, Apgar score, and systemic side effects. A third investigator cross-checked the extracted data to resolve any discrepancies.

Our primary outcomes are Recurrent uterine contractions, Systemic side effects, Latency period, Pregnancy complications, Cesarean section incidence, and Gestational Age at delivery.

Pregnancy complications were defined as any clinically significant maternal condition occurring during pregnancy or the postpartum period that required medical assessment or management, including infectious (e.g., endometritis, wound infection), obstetric (e.g., postpartum hemorrhage), or psychiatric events (e.g., postpartum psychosis).

Systemic side effects were defined as non-obstetric adverse events temporally associated with dydrogesterone administration affecting systemic physiological functions, such as cardiovascular (tachycardia), gastrointestinal (nausea/vomiting), or metabolic disturbances (hypokalemia), consistent with known progestin-related reactions.

Two investigators assessed the quality of the studies using the Cochrane Collaboration’s Risk of Bias Tool (ROB1). Studies were graded as “low risk,” “unclear,” or “high risk” based on the following seven domains: random sequence generation, allocation concealment, blinding of participants, blinding of outcome assessment, incomplete outcome data, selective reporting, and other sources of bias.

### Statistical analysis

Statistical analyses were conducted using R software (version 4.5.0) on a Windows platform (x86_64-w64-mingw32). We employed the *meta* package (version 8.2.0) for performing the meta-analyses and generating statistical visualizations. We evaluated the dichotomous data using risk ratios and continuous data using mean differences with 95% confidence intervals. If the data exists as median and IQR, we convert it to mean and SD using Xiang Wan et al.’s Formula [21]. Heterogeneity was reported using I². An I² value between 0% and 40% indicates that heterogeneity is not important, while values between 30% and 60% suggest moderate heterogeneity. Similarly, I² values ranging from 50% to 90% are generally interpreted as representing substantial heterogeneity, and those between 75% and 100% suggest considerable heterogeneity.

A fixed-effect model was used when heterogeneity was low (I² ≤50%), whereas a random-effects model was applied when significant heterogeneity was detected (I² >50%). To assess the influence of individual studies on the overall results and the extent of heterogeneity, we performed a leave-one-out sensitivity analysis, in which each study was excluded sequentially. This allowed us to determine whether any single study had a disproportionate impact on the pooled effect estimate or the level of heterogeneity. Subgroup meta-analyses were conducted to explore sources of heterogeneity, including subgrouping by daily dydrogesterone dose (two vs. three tablets) and by study country.

We also performed a trial sequential analysis (TSA) to quantitatively evaluate whether additional studies with larger sample sizes are necessary. This analysis was conducted using the Trial Sequential Analysis software (Version 0.9.5.10 Beta, Copenhagen Trial Unit, Centre for Clinical Intervention Research, The Capital Region, Copenhagen University Hospital – Rigshospitalet, 2021). The TSA was carried out using standard parameters, including 5% Type I error rate, 80% statistical power, the O’Brien-Fleming alpha-spending function, variance-based adjustment for heterogeneity, and empirical estimates for the mean difference and variance.

### Certainty assessment

The quality of evidence was evaluated using the GRADE (Grading of Recommendations Assessment, Development and Evaluation) methodology. Two reviewers independently completed the assessment using the GRADEpro Guideline Development Tool [Software], developed by McMaster University and Evidence Prime (2025), available at gradepro.org. Any disagreements were resolved through discussion or, if needed, involving a third reviewer. For each primary outcome, the certainty of the evidence was rated as high, moderate, low, or very low, considering five key GRADE criteria: risk of bias, inconsistency, indirectness, imprecision, and publication bias. A summary of the findings is presented in Table 2.

## Results

### Study selection

**Fig. 1:**
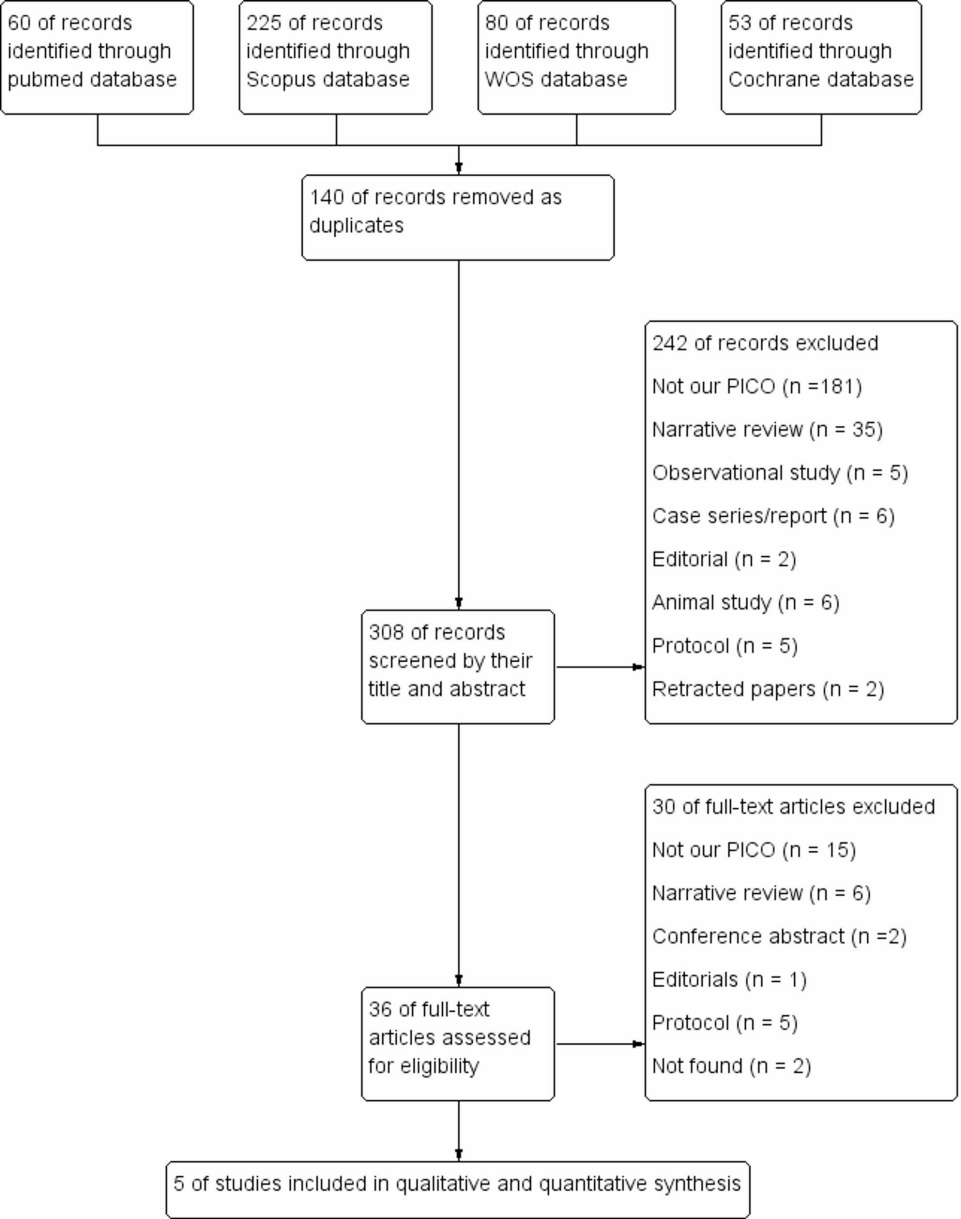
The PRISMA flow diagram of studies screening and selection

The electronic search identified 364 records, 110 of which were duplicates and were removed using EndNote software. The remaining 254 records were screened by title and abstract, and 219 were excluded. The remaining 35 records were screened in full text, and only 5 clinical trials matched our inclusion criteria and 30 records were excluded. Details of the selection process and reasons for exclusion are illustrated in Fig. 1.

### Characteristics of included studies

Five studies with 436 patients were included in our study; 218 in each of the intervention and control groups. The mean age of all patients was 31.98, and it was 29.55 and 28.4 for the dydrogesterone and control groups, respectively. The rest of the baseline characteristics are reported in Table 1. Some studies included groups rather than dydrogesterone and control, but we didn’t include them in our analysis as another comparison because of insufficient data. Our main outcomes were the incidence of recurrent uterine contractions, latency period, gestational age, pregnancy complications, C-sections, and systemic side effects. Other outcomes related to neonates are also analyzed, including the following: Apgar score, low birth weight, incidence of IVH, NICU admission, NEC, sepsis, apnea of prematurity, TTNB, mortality, RDS, and neonatal birth weight.

**Table 1:** Study characteristics

Study	Country	Aim of the study	Primary Outcomes	Conclusion of the most important results	Adjacent treatment	Treatment groups(*n*)	Maternal Age at Admission	Primigravida *n*(total)	Nulliparous	GA (weeks) (Mean ± SD)	BMI (Mean ± SD)	History of preterm *n*(total)
Alizadeh et al., 2022^a^ [38]	Iran	To assess DG in terms of reducing the incidence of PTB.	Occurrence of PTB, latency period, and neonatal outcomes (birth weight, Apgar score, NICU admission).	oral DG wasn’t as effective in preventing PTB, prolonging the latency period, and neonatal outcomes.	Tocolytic treatment	DG (55) No-intervention (55)	28.6 ± 4.04 28.71 ± 3.98	NA	NA	31.5 ± 1.9 31.72 ± 1.83	NA	NA
Areeruk et al., 2016 [39]	Thailand	To evaluate the effect of oral DG on the recurrent uterine contraction in PTB.	Rate of uterine contraction.	Oral DG 20 mg/day could not decrease the rates of recurrent uterine contraction and prolong the latency period in PTB management compared to placebo.	Tocolytic treatment	DG (24) Placebo (24)	31 ± 6.3 27.7 ± 6.9	7(24) 10(24)	9(24) 11(24)	32.3 ± 1.6 31.4 ± 2.4	21.4 ± 2.4 21.5 ± 3.4	3(24) 2(24)
Keshtmandi et al., 2023 [40]	Iran	To assess the effectiveness of oral DG on maternal and neonatal results in treating PTB.	Incidence of preterm delivery, Recurrent uterine contraction, gestational age at delivery, latency period, and Apgar score.	Oral DG adjuvant to MgSO4 was ineffective in preventing PTB, improving pregnancy outcomes, or reducing uterine contraction recurrence. However, the 1-minute Apgar score improved compared to the placebo group.	MgSO4	DG (50) Placebo (50)	29.2 ± 4 28±3.5	18(50) 13(50)	NA	NA	22.1 ± 2.2 22.3 ± 2.4	5(50) 4(50)
Thongchan et al., 2021 [41]	Thailand	To evaluate the efficacy of oral DG on latency period in managing PTB.	Latency period	Adjunctive treatment with 30 mg of oral DG could not prolong the latent period in preterm labor compared to placebo.	Tocolytic treatment	DG (24) Placebo (24)	29.9 ± 5.7 29.2 ± 7.6	13(24) 12(24)	14(24) 17(24)	31.5 ± 2.1 31.4 ± 2.3	22.5 ± 4.7 22.2 ± 5.9	2(24) 1(24)
Lotfalizadeh et al., 2019^b^ [42]	Iran	To compare the duration of pregnancy in the administration of Duphaston tablets in pregnant women with preterm labor after stopping the delivery process.	The increase in the duration of pregnancy from the start of treatment to delivery, gestational age at delivery, and neonatal results, including birth weight, Apgar score, and NICU hospitalization.	Duphaston tablets increased the duration of pregnancy, the age of pregnancy termination, neonatal weight, and the mean of 1- and 5-minute Apgar scores, which were less effective in increasing neonatal weight.	Tocolytic treatment and MgSo4	DG (65) Control (65)	29.94 ± 3.4 NA	NA	NA	31.69 ± 5.93 31.69 ± 7.3	NA	NA

*DG* Dydrogesterone, *17*α-*OHPC* 17α-hydroxyprogesterone caproate, *GA* Gestational age, *PTB* Preterm birth, *BMI* Body mass index, *MgSO4* Magnesium Sulfate, Maternal age, GA, and BMI are presented in mean ± SD, Primigravida, Nulliparous, history of preterm, and stillbirth are presented in event (total)

^a^One group was excluded from the meta-analysis as it included 17α-hydroxyprogesterone caproate

^b^One group was excluded from the meta-analysis as it included Cyclogest

### Quality assessment and quality of evidence

**Fig. 2:**
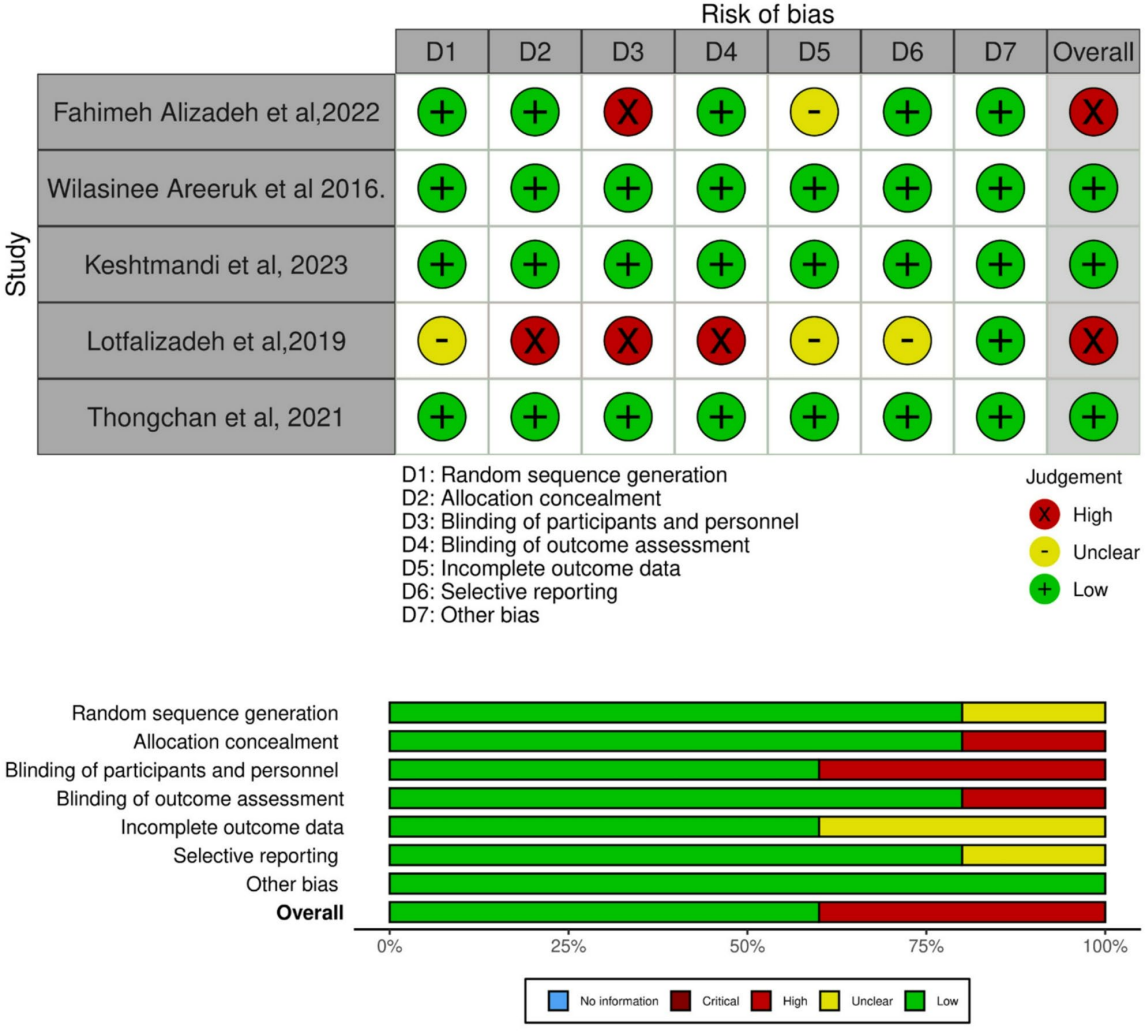
A risk of bias analysis of the included studies in our systematic review and meta-analysis.” +” indicates low risk;”-” indicates unclear and” X” indicates high

The quality assessment was conducted using the Cochrane Collaboration’s Risk of Bias tool (ROB 1). Out of the five studies, three had a low risk of bias, and two had a high risk of bias; details of quality assessment are presented in Fig. 2.

The quality of evidence for the main outcomes was assessed using the GRADE approach and ranged from moderate to very low. A detailed summary of the primary outcomes according to GRADE guidelines is presented in Table 2.

**Table 2:** Summary of findings (SoF) of the main outcomes according to GRADE assessment

Outcomes	Number of studies	Number of patients		Effect		Certainty
		dydrogesterone	placebo	Relative (95% CI)	Absolute (95% CI)	
Recurrent uterine contractions	2	27/74 (36.5%)	66/74 (89.2%)	RR 0.38 (0.01 to 11.08)	553 fewer per 1,000 (from 838 fewer to 1,000 more)	⨁◯◯◯ Very low^a, b,c^
Systemic side effects	2	4/74 (5.4%)	0/74 (0.0%)	RR 9.00 (0.51 to 158.35)	0 fewer per 1,000 (from 0 fewer to 0 fewer)	⨁⨁◯◯ Low^b, c,e^
Latency period	3	98	98	-	MD 3.71 days higher (12.93 lower to 20.35 higher)	⨁◯◯◯ Very low^a, b,c, d^
Pregnancy complications	3	6/98 (6.1%)	5/98 (5.1%)	RR 1.15 (0.42 to 3.18)	8 more per 1,000 (from 30 fewer to 111 more)	⨁⨁⨁◯ Moderate^b, c^
Cesarean section incidence	4	64/148 (43.2%)	80/148 (54.1%)	RR 0.80 (0.64 to 1.01)	108 fewer per 1,000 (from 195 fewer to 5 fewer)	⨁⨁◯◯ Low^c, d^
Gestational Age at delivery	5	213	213	-	MD 0.42 weeks higher (0.7 lower to 1.55 higher)	⨁◯◯◯ Very low^a, c,d^

*CI* Confidence interval, *MD* Mean difference, *RR* Risk ratio

^a^high heterogeneity, ^b^small sample size, ^c^Confidence interval includes both benefit and harm, ^d^some study has high RoB, ^e^Very large confidence interval

### Analysis

#### Maternal outcomes

**Fig. 3:**
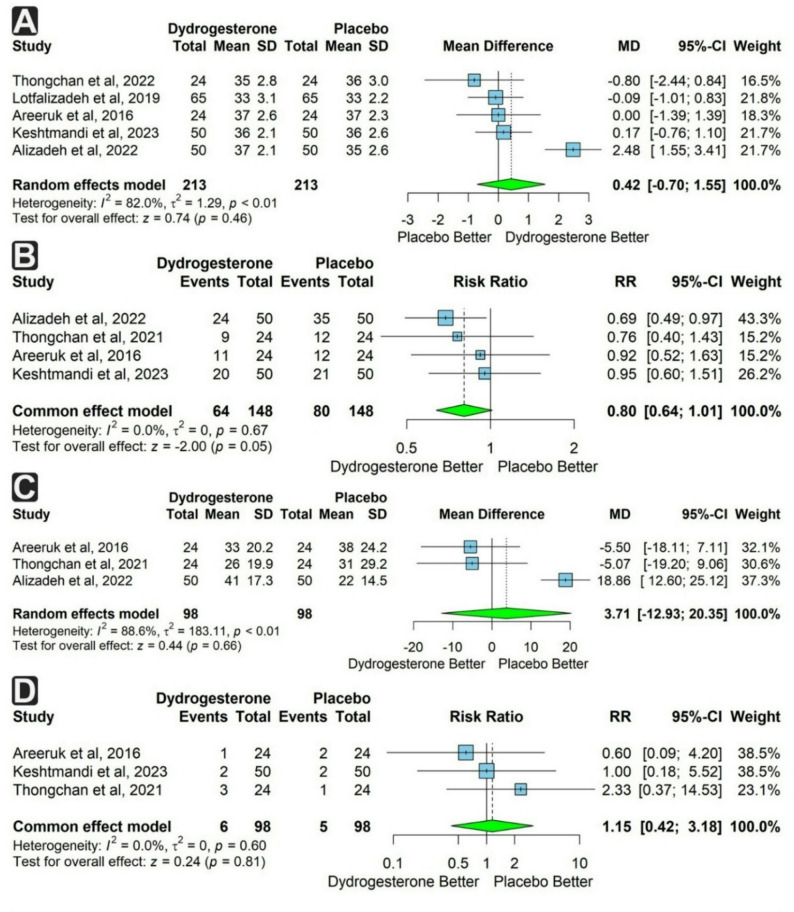
Forest plots of maternal outcomes comparing dydrogesterone versus placebo. **A** Mean difference in gestational age at delivery. **B** Risk of recurrent cesarean section. **C** Mean difference in latency period. **D** Risk of pregnancy complications

Five studies reported the gestational age (GA) at delivery, with a total of 426 participants, 213 in each group. The dydrogesterone group showed a non-significant trend toward a higher GA compared to the placebo group (mean difference [MD] = 0.42 weeks; 95% confidence interval [CI]: −0.70 to 1.55; *p* = 0.46). Substantial heterogeneity was observed (I² = 82%) (Fig. 3a).

We conduct subgroup analysis based on the number of dydrogesterone tablets taken per day and based on the study country. The three tablets subgroup demonstrated significant heterogeneity (I² = 88.5%), and the effect remained non-significant. The Thailand subgroup demonstrated no heterogeneity (I² = 0%), whereas the Iran subgroup retained high heterogeneity (I² = 88.9%). However, the effect remained non-significant in both subgroups (Supplementary Figs. 1 & 2).

Four studies reported the incidence of caesarean section (CS), with 296 participants in total (148 in each group). The rate of CS was non-significantly lower in the dydrogesterone group (risk ratio [RR] = 0.80; 95% CI: 0.64 to 1.01; *p* = 0.05), with no heterogeneity detected (I² = 0%) (Fig. 3b).

Three studies reported the latency period and pregnancy complications among 196 participants (98 in each group). The latency period was longer in the dydrogesterone group, but the difference was not statistically significant (MD = 3.71 days; 95% CI: −12.93 to 20.35; *p* = 0.66). Substantial heterogeneity was observed (I² = 88.6%). There was no significant difference in the incidence of pregnancy complications (RR = 1.15; 95% CI: 0.42 to 3.18; *p* = 0.81), with no heterogeneity observed (I² = 0%) (Fig. 3c & d).

**Fig. 4:**
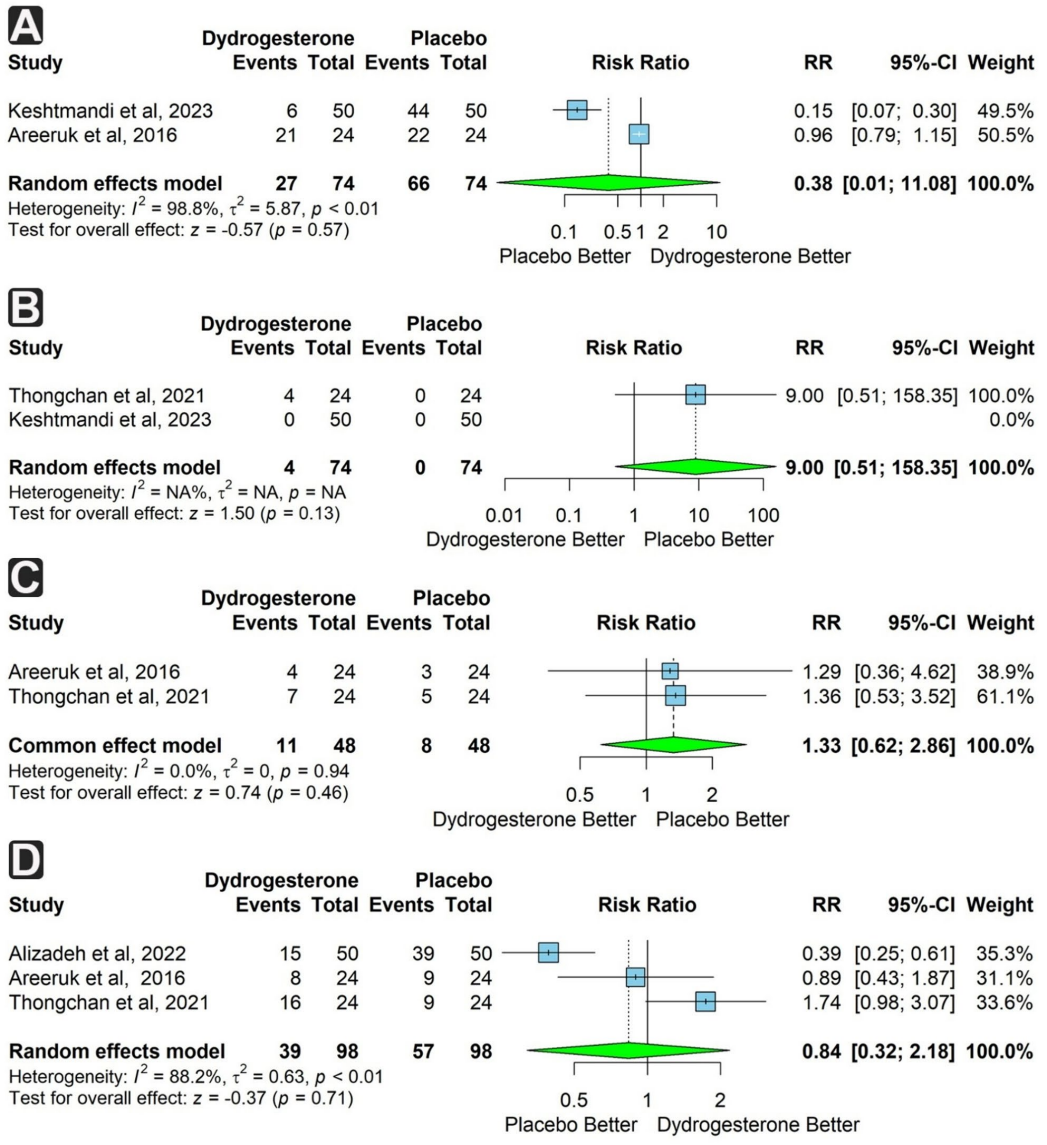
Forest plots of maternal outcomes comparing dydrogesterone versus placebo. **A** Risk of recurrent uterine contractions. **B** Risk of systemic side effects. **C** Risk of gestational age at delivery < 34 weeks. **D** Risk of gestational age at delivery < 37 weeks

Two studies assessed the incidence of recurrent uterine contractions and systemic side effects in 148 participants (74 in each group). Both outcomes were not significantly different between groups (RR = 0.38; 95% CI: 0.01 to 11.08; *p* = 0.57 and RR = 9.00; 95% CI: 0.51 to 158.35; *p* = 0.13, respectively). Heterogeneity was substantial for uterine contractions (I² = 98.8%) (Fig. 4a & b).

Two studies reported the incidence of delivery at GA < 34 weeks, with 96 total participants (48 per group). This outcome was more frequent in the dydrogesterone group but not significantly so (RR = 1.33; 95% CI: 0.62 to 2.86; *p* = 0.46), with no heterogeneity (Fig. 4c). Three studies reported delivery at GA < 37 weeks (196 participants, 98 per group). The incidence was lower in the dydrogesterone group, though not significantly (RR = 0.84; 95% CI: 0.32 to 2.18; *p* = 0.71), with substantial heterogeneity (I² = 88.2%) (Fig. 4d).

#### Neonatal outcomes

**Fig. 5:**
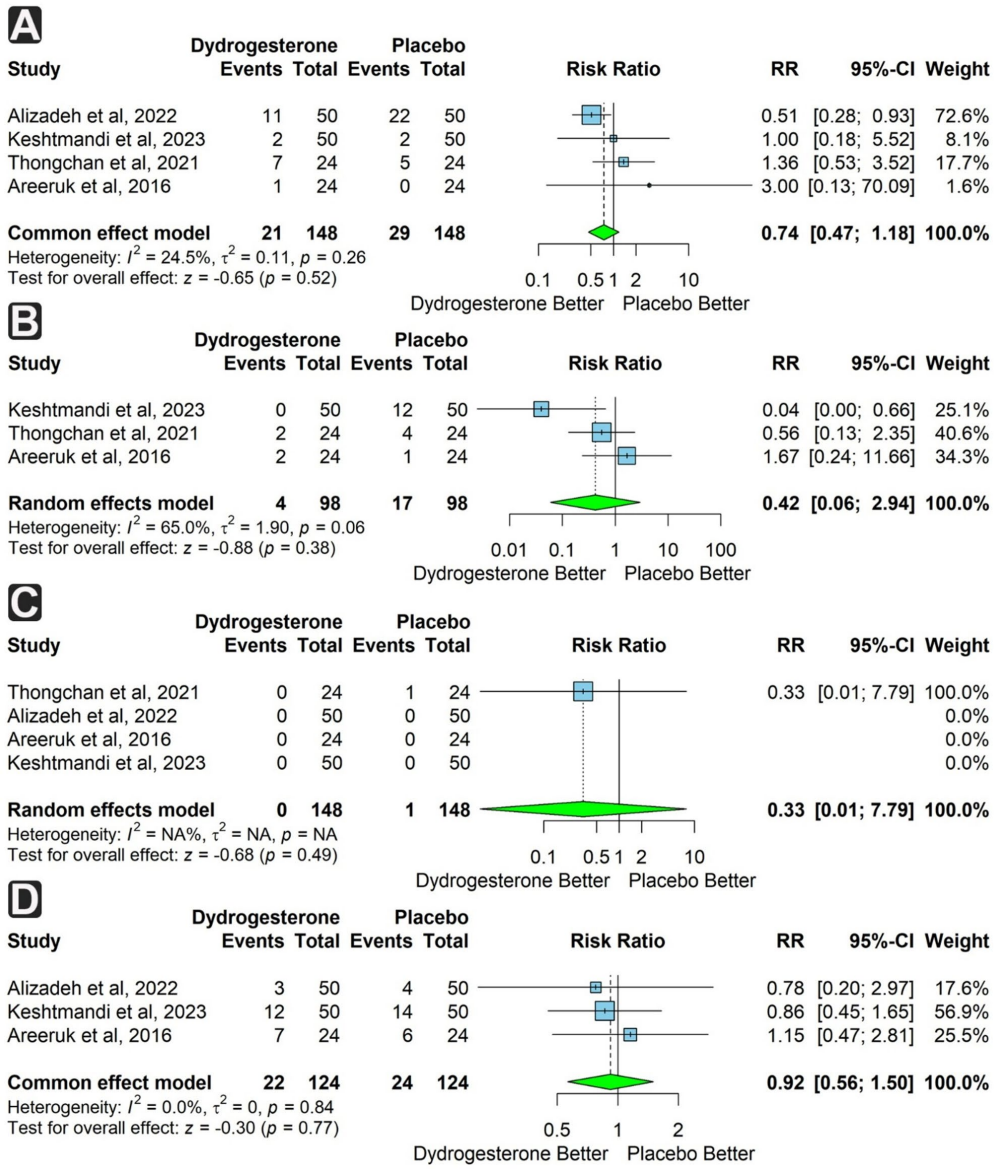
Forest plots of neonatal outcomes comparing dydrogesterone versus placebo. **A** Risk of neonatal intensive care unit (NICU) admission. **B** Risk of Apgar score < 7 at 1 min. **C** Risk of Apgar score < 7 at 5 min. **D** Risk of low birth weight

Four studies (296 participants, 148 per group) assessed NICU admission. The rate was lower in the dydrogesterone group but not significantly (RR = 0.74; 95% CI: 0.47 to 1.18; *p* = 0.52), with low heterogeneity (I² = 24.5%) (Fig. 5a).

Three studies (196 participants, 98 per group) assessed Apgar score < 7 at 1 min. The risk was lower in the dydrogesterone group (*n* = 4) compared to the control (*n* = 17), but not significantly (RR = 0.42; 95% CI: 0.06 to 2.94; *p* = 0.38). Moderate heterogeneity was present (I² = 65%). Four studies (296 participants, 148 per group) assessed Apgar score < 7 at 5 min. Although more cases were observed in the placebo group, the risk difference was not significant (RR = 0.33; 95% CI: 0.01 to 7.79; *p* = 0.49). Heterogeneity was not assessed (Fig. 5b & c).

Three studies (248 participants, 124 per group) reported low birth weight. The outcome was less frequent in the dydrogesterone group, but not significantly (RR = 0.92; 95% CI: 0.56 to 1.50; *p* = 0.77), with no heterogeneity (I² = 0%) (Fig. 5d).

**Fig. 6:**
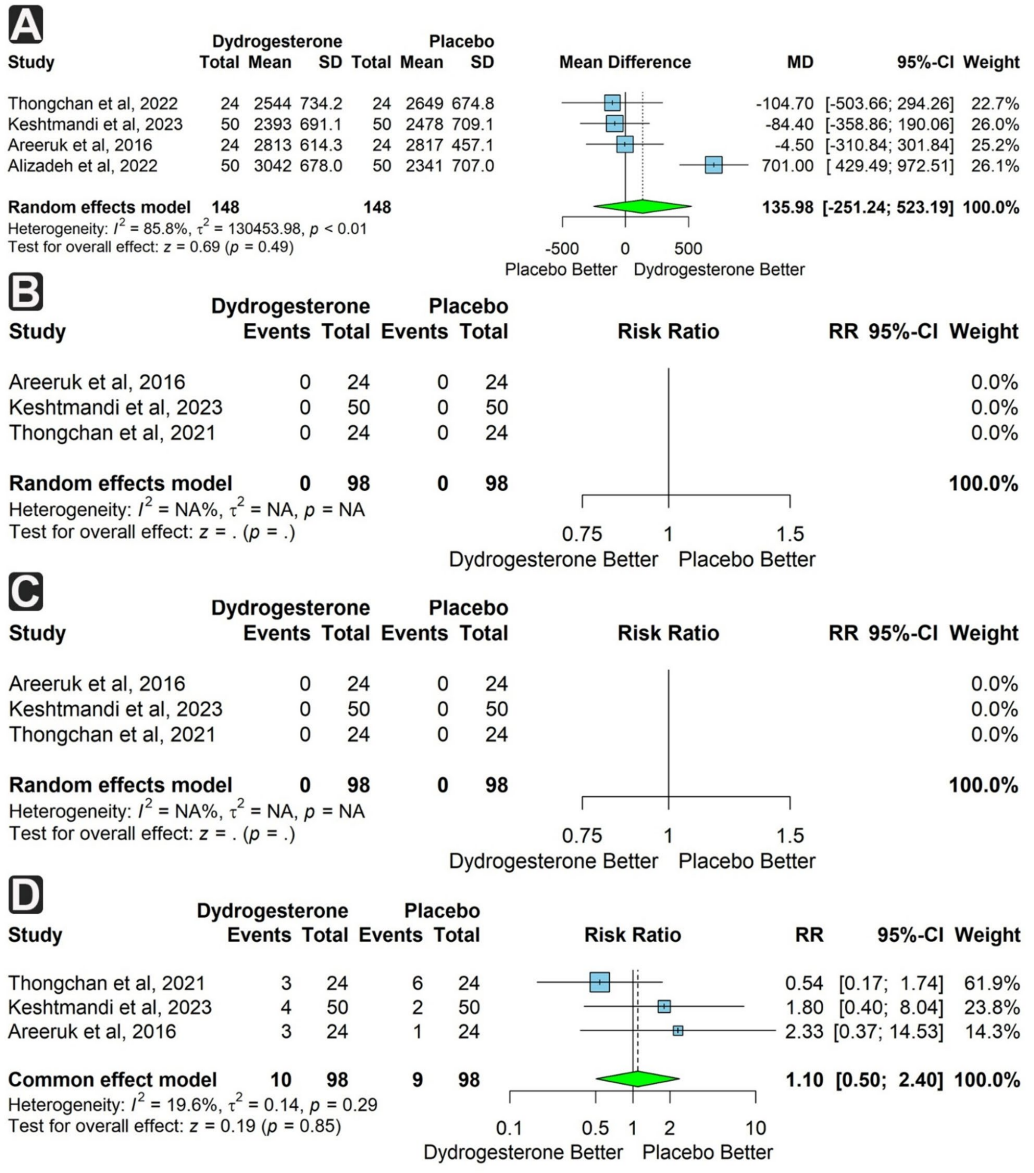
Forest plots of neonatal outcomes comparing dydrogesterone versus placebo. **A** Mean difference in birth weight (grams). **B** Risk of intraventricular hemorrhage (IVH). **C** Risk of necrotizing enterocolitis (NEC). **D** Risk of neonatal sepsis

Four studies (296 participants, 148 per group) reported birth weight in grams. The dydrogesterone group showed a higher mean birth weight, though not significant (MD = 135.98 g; 95% CI: −251.24 to 523.19; *p* = 0.49), with substantial heterogeneity (I² = 85.8%) (Fig. 6a).

Subgroup analysis by number of dydrogesterone tablets taken per day showed significant heterogeneity within the three tablet subgroups (I² = 89.6%), with no statistical significance. Subgroup analysis by study country demonstrated the absence of heterogeneity within the Thailand subgroup (I² = 0%), whereas the Iran subgroup showed markedly increased heterogeneity (I² = 93.7%). Despite these differences, neither subgroup reached statistical significance (Supplementary Figs. 3 & 4).

Three studies (196 participants) reported the incidence of IVH and NEC. No events were reported in either group (Fig. 6b & c).

**Fig. 7:**
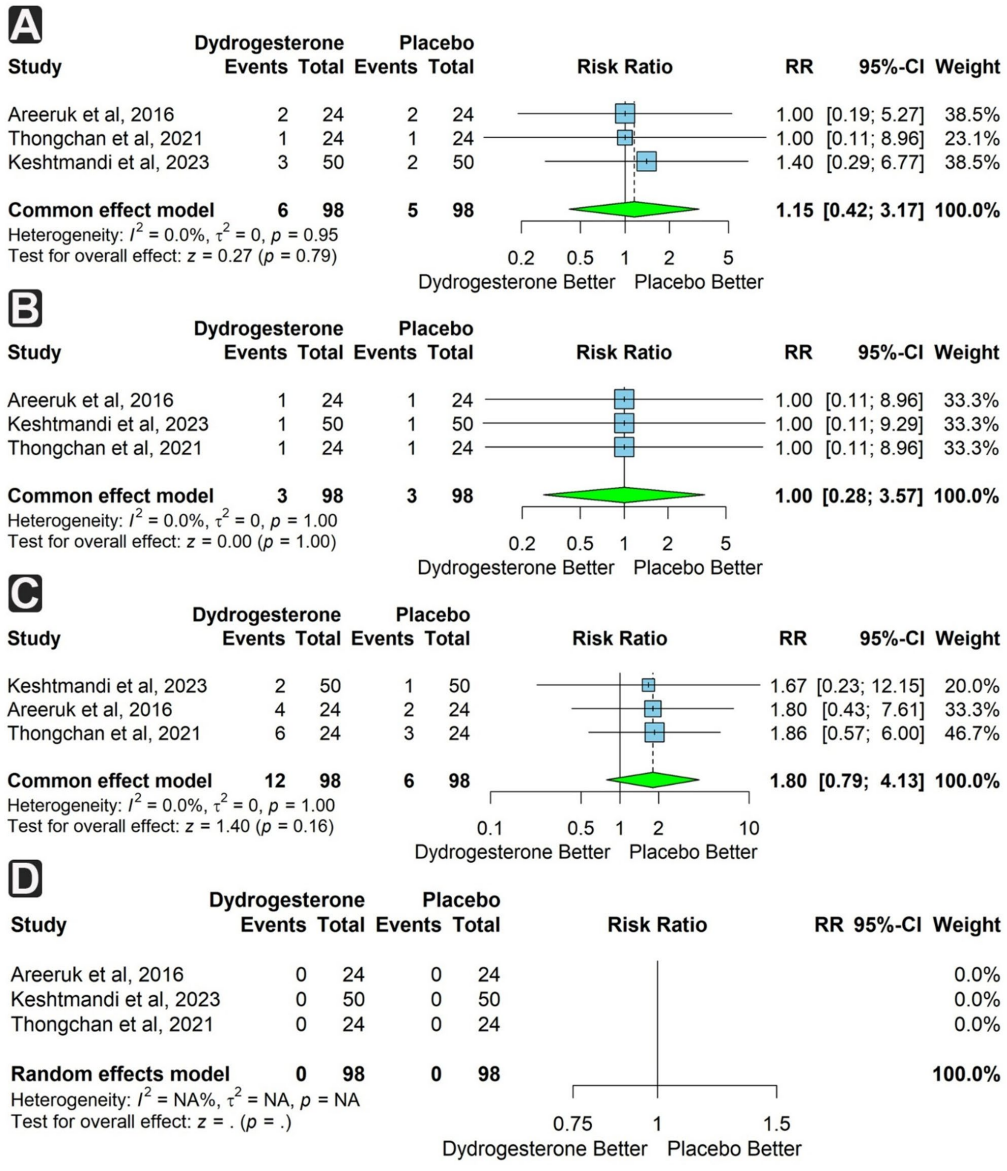
Forest plots of neonatal outcomes comparing dydrogesterone versus placebo. **A** Risk of apnea of prematurity. **B** Risk of transient tachypnea of the newborn. **C** Risk of respiratory distress syndrome (RDS). **D** Risk of neonatal mortality

Three studies (196 participants) assessed neonatal sepsis and apnea of prematurity. Both outcomes were more frequent in the dydrogesterone group, but not significantly (RR = 1.10; 95% CI: 0.50 to 2.40; *p* = 0.85 and RR = 1.15; 95% CI: 0.42 to 3.17; *p* = 0.79, respectively). Heterogeneity was low for sepsis (I² = 19.6%) and absent for apnea (I² = 0%) (Figs. 6d and 7a).

Three studies (196 participants) reported transient tachypnea of the newborn (TTN), respiratory distress syndrome (RDS), and neonatal mortality. TTN was equally reported in both groups (RR = 1.00; 95% CI: 0.28 to 3.57; *p* = 1). RDS was more frequent in the dydrogesterone group, though not significantly (RR = 1.8; 95% CI: 0.79 to 4.13; *p* = 0.16). No neonatal deaths occurred in either group. No heterogeneity was found for these outcomes (Fig. 7b & d).

#### Leave-one-out meta-analysis

We conducted a leave-one-out meta-analysis to explore sources of heterogeneity and assess the influence of each individual study on the overall results.

Excluding Alizadeh et al. (2022) from the latency period analysis resulted in a substantial reduction in heterogeneity from I² = 88.6% to I² = 0.0%. However, the mean difference (MD) remained statistically non-significant, changing from 3.71 days (95% CI: −12.93 to 20.35) to −5.31 days (95% CI: −14.72 to 4.10). Similar trends were observed when the same study was removed from the birth weight analysis, where heterogeneity decreased from I² = 85.8% to I² = 0.0%, and the MD shifted from 136 g (95% CI: −251.24 to 523.19) to −60.44 g (95% CI: −242.37 to 121.48), without altering the statistical significance (Supplementary Figs. 5 & 6).

In the gestational age at delivery (weeks) outcome, removing Alizadeh et al. led to a reduction in heterogeneity from I² = 82.0% to I² = 0.0%. The MD changed from 0.42 weeks (95% CI: −0.70 to 1.55) to −0.06 weeks (95% CI: −0.62 to 0.49), remaining non-significant. For preterm birth < 37 weeks, heterogeneity decreased from I² = 88.2% to I² = 49.2%, and the pooled RR changed from 0.84 (95% CI: 0.32–2.18) to 1.3 (95% CI: 0.68–2.49), also without reaching statistical significance (Supplementary Figs. 7 & 8).

Interestingly, removal of Keshtmandi et al. (2023) for the cesarean section outcome affected the effect estimate, which shifted from statistically non-significant (RR = 0.8; 95% CI: 0.64–1.01; *p* = 0.066) to significant (RR = 0.75; 95% CI: 0.58–0.98; *p* = 0.036) (Supplementary Fig. 9). Finally, removal of Keshtmandi et al. (2023) from the analysis of Apgar score < 7 at 1 min reduced heterogeneity from I² = 65% to I² = 0.0%. The pooled RR increased from 0.42 (95% CI: 0.06–2.94) to 0.82 (95% CI: 0.26–2.61), with both estimates remaining statistically nonsignificant (Supplementary Fig. 10).

#### Trial sequential analysis

Trial sequential analysis (TSA) indicated that additional studies including at least 6,276 patients are required to produce reliable and conclusive evidence regarding gestational age at delivery. As illustrated in Supplementary Fig. 11, the cumulative Z-curve (blue line) does not cross any of the trial sequential monitoring boundaries (red lines), demonstrating that the currently available evidence is insufficient to confirm or exclude a true effect of dydrogesterone. Moreover, the Z-curve does not reach the required information size (vertical red line), signifying that the accumulated sample size remains well below what is necessary for robust inference. Collectively, the TSA findings visually and statistically highlight that existing trials are underpowered and that substantially larger, high-quality studies are still needed to establish firm conclusions.

## Discussion

In this systematic review and meta-analysis, we synthesized evidence on the efficacy of dydrogesterone in the prevention or treatment of preterm birth compared to placebo. Overall, the meta-analysis results revealed no significant differences in maternal or neonatal outcomes between the dydrogesterone and placebo groups in all outcomes.

The observed reduction in cesarean section rates and numerically favorable point estimates for gestational age at delivery, NICU admission, and Apgar scores did not reach statistical significance. Therefore, these findings should be interpreted with caution and cannot be considered evidence of a clinically meaningful benefit. Nevertheless, they may suggest potential signals that warrant further investigation, particularly in selected patient populations. Subgroups such as women with a history of preterm birth or those with a sonographically short cervix may derive differential benefit, a hypothesis that remains untested in adequately powered randomized trials. In such high-risk populations, even modest improvements in gestational age or neonatal outcomes could be clinically relevant. This cautious interpretation is informed by prior evidence demonstrating the efficacy of progesterone in reducing preterm birth among women with a short cervix, as reported by Fonseca et al. (2007), Hassan et al. (2011), and Romero et al. (2012), which showed a reduction in preterm birth following treatment with vaginal progesterone [22–24].

Importantly, the non-significant trends observed across the outcomes were small in magnitude and not clinically meaningful, and therefore do not support replacing established vaginal or injectable progesterone regimens with oral dydrogesterone.

These findings also mirror the broader inconsistency observed in the literature regarding various forms of progesterone therapy. For micronized progesterone (Noblot et al., 1991) and Dodd et al., 2013, both found no significant benefit in preventing preterm delivery [25, 26].

Similarly, studies investigating daily administration of 200 mg vaginal progesterone, such as those by Martinez De Tejada et al. (2015 and Norman et al. (2009 reported no significant effect [27, 28]. Eke et al. (2016 also found no significant difference across different routes and formulations, including 17-alpha hydroxyprogesterone caproate (17-OHPC), vaginal, and micronized progesterone [29]. Similarly, studies evaluating IM 17-OHPC, such as those by Ibrahim et al., 2010, and Gupta et al., indicated potential benefits in reducing recurrent preterm birth [30]. Moreover, Gupta S et al. said that the use of 17 alpha hydroxy progesterone could be useful in pregnant females with a history of preterm delivery to decrease the risk of recurrence [31].

Many of the included studies contributed to the observed heterogeneity, particularly when individual studies were excluded in sensitivity analyses. However, the interpretation of heterogeneity in this review is inherently limited by the small number of available trials (*n* = 5), as heterogeneity metrics such as I² are statistically unstable and often unreliable when the number of studies is low [32]. Furthermore, most trials did not report sufficient key baseline categorical data, which limited our ability to perform some warranted subgroup meta-analyses.

Despite this limitation, we conducted a subgroup analysis based on the daily dydrogesterone dose: three studies reported administering three tablets per day (Thongchan et al., 2022; Keshtmandi et al., 2023; Alizadeh et al., 2022), while two studies used a regimen of two tablets per day (Areeruk et al., 2016; Lotfalizadeh et al., 2019).

We also observed that the two studies that most strongly influenced the sensitivity analyses (Alizadeh et al. and Keshtmandi et al.) were both conducted in Iran. This may reflect population-specific factors such as genetic background or different baseline risk profiles. In addition, Alizadeh et al. were rated at high risk of bias due to a lack of blinding, which may also have contributed to inconsistent findings.

Based on these findings, we performed an exploratory subgroup analysis by study country. While this helped illustrate patterns in heterogeneity (with I² = 0% in Thailand and I² = 88–94% in Iran), the results remained statistically non-significant in all subgroups.

Given that only five studies were available, these subgroup analyses should be considered exploratory and interpreted with caution. With such a small evidence base, subgroup findings are prone to false-positive or false-negative patterns and cannot be considered confirmatory [33].

Major international guidelines consistently recommend progesterone supplementation only for well-defined high-risk groups, and they restrict their recommendations to vaginal or injectable formulations. The FIGO 2021 good-practice recommendations advise the use of daily vaginal progesterone or weekly 17-OHPC for women with a previous spontaneous preterm birth or a very short cervix [34]. Likewise, NICE recommends prophylactic vaginal progesterone for women who have both a history of preterm birth and a short cervix. Global health agencies echo this approach [35]. The WHO preterm-birth framework emphasizes evidence-based interventions such as antenatal corticosteroids and tocolytics, and does not endorse routine progestogen use outside high-risk cases [36]. Notably, none of these major bodies include oral dydrogesterone in their recommendations, as current guidelines are based on large, well-designed trials involving other progestogen formulations.

Within this context, our meta-analysis—which demonstrated no statistically significant benefit of oral dydrogesterone in preventing preterm birth or improving maternal or neonatal outcomes—reinforces adherence to existing guideline-supported regimens that recommend only vaginal or injectable progesterone for well-defined high-risk groups. Although we observed favorable but non-significant trends in gestational age (MD ≈ 0.42 weeks), cesarean section, and NICU admission, these effects are too small to be clinically meaningful; an additional three days of gestation rarely translates into reduced neonatal morbidity outside extremely high-risk scenarios, and the modest reductions in other outcomes fall within expected random variation in small trials. Therefore, oral dydrogesterone should not replace established progesterone strategies, and any reconsideration of its clinical role will require rigorously designed, adequately powered randomized trials.

### Strengths & limitations

This is the first systematic review and meta-analysis in our knowledge that highlights the efficacy of oral dydrogesterone in preterm management and its maternal and neonatal effects. We exclusively included RCTs to ensure the accuracy of our data and reach the highest level of evidence. In Fig. 1, we illustrate the reason for the exclusion from other studies and cross-check every step to ensure the reliability of our work. In addition, we assessed the quality of evidence according to the GRADE guidelines and TSA to know quantitatively if we need more studies with a larger sample size. Moreover, we conducted a leave-one-out meta-analysis to ensure that no single study disproportionately influenced the results. We also examined a wide range of outcomes, such as recurrence of uterine contractions, latency period, systemic and maternal side effects, and neonatal complications such as NICU admission, NEC, RDS, etc.

Our research has some drawbacks too, such as the small number of studies and total patients, which resulted in insufficient data for certain groups, so we couldn’t put other comparisons in the meta-analysis. All studies were conducted in two countries (Iran and Thailand), which restricts generalizability to other populations, including high-income settings or women with multiple gestations, medical comorbidities, or differing obstetric practices.

Another limitation is that some studies have poor quality, and we didn’t exclude them from analysis because of the limited number of studies; So, it may affect the evidence. In addition, assessment of publication bias was not performed because only five trials were included, and according to Cochrane recommendations, funnel plot–based methods and statistical tests for small-study effects are unreliable and underpowered when fewer than ten studies are available [37].

Although our results do not indicate statistically significant differences in most outcomes, the generalization of the results is doubtful. Quality of evidence by GRADE revealed that most of the outcomes have low or very low evidence; in addition, trial sequential analysis shows that current evidence is substantially underpowered, estimating that 6,276 participants would be required to reach a firm conclusion, and we need more high-quality studies with larger sample sizes to confirm the evidence.

To more effectively evaluate the benefits of oral DG as a prevention method for preterm labor, a future high-quality randomized controlled trial with a larger participant pool should be conducted. Furthermore, other routes and dosages of dydrogesterone should be studied and compared to different types of progesterone to draw a precise conclusion about the overall efficacy of progesterone in preventing preterm delivery.

## Conclusion

Although the use of dydrogesterone in the prevention of preterm in some countries, our systematic review and meta-analysis show that dydrogesterone demonstrated non-significant trends toward improved outcomes when compared with placebo in preventing preterm delivery or improving maternal or neonatal outcomes. Most outcomes were rated as low or very low certainty using GRADE, and TSA confirmed that the current evidence base is insufficient to support clinical recommendations. Therefore, dydrogesterone cannot be recommended for routine prevention of preterm birth, and we need more high-quality RCTs to emphasize the evidence.

## Supplementary Information

Supplementary Material 1.

Supplementary Material 2.

## Data Availability

The data extraction sheets, statistical analysis files, and other materials used in this systematic review and meta-analysis are available from the corresponding author upon request.

## Abbreviations

PTL: Preterm labor
PTB: Preterm birth
IVH: Intraventricular hemorrhage
RDS: Respiratory distress syndrome
NEC: Necrotizing enterocolitis
GA: Gestational age
NICU: Neonatal intensive care unit
BP: Blood pressure
ROB 1: Risk of Bias 1
