# Supplementary material for: Effectiveness of oral dydrogesterone compared to placebo in reducing the risk of preterm birth: a systematic review and meta-analysis
Source: BMC Pregnancy Childbirth. 2026 Feb 24;26:222. doi: 10.1186/s12884-026-08747-5 (PMC12955320; doi:10.1186/s12884-026-08747-5)
Supplement: Supplementary file 1 — Supplementary Material 1. [file 12884_2026_8747_MOESM1_ESM.docx]

**Supplementary Material**

***Title:*** **Effectiveness of Oral Dydrogesterone Compared to Placebo in Reducing the Risk of Preterm Birth: Systematic Review and Meta-Analysis**

***Authors:*** Sohieb Hedawy*^1^, Shahed Aldalahmeh^2^, Eman E. Labeeb^3^, Abdelrahman A. Khattab^3^, Esraa K. Sayed^4^, Ahmed Hassan^5^, Ahmed Menshawy^6^

| **Supplementary Content:** | | |
| --- | --- | --- |
| **a. Tables:** | | **Page** |
| **1** | **Table S1**. Detailed search strategy. | **2** |
| **b. Figure:** | | **Page** |
| **1** | **Supplementary Fig 1:** Subgroup Analysis by Tablets for gestational age at delivery (Three Tablets vs. Two Tablets). | **2** |
| **2** | **Supplementary Fig 2:** Subgroup Analysis by Country for gestational age at delivery (Thailand vs. Iran). | **3** |
| **3** | **Supplementary Fig 3:** Subgroup Analysis by Tablets for Birth Weight in grams (Three Tablets vs. Two Tablets). | **3** |
| **4** | **Supplementary Fig 4:** Subgroup Analysis by Country for Birth Weight in grams (Thailand vs. Iran). | **4** |
| **5** | **Supplementary Fig 5:** Leave one out meta-analysis of latency period after exclusion of Alizadeh et al, 2022, dydrogesterone vs. placebo | **4** |
| **6** | **Supplementary Fig 6:** Leave one out meta-analysis of birth weight in grams after exclusion of Alizadeh et al, 2022, dydrogesterone vs. placebo | **5** |
| **7** | **Supplementary Fig 7:** Leave one out meta-analysis of GA at delivery in weeks after exclusion of Alizadeh et al, 2022, dydrogesterone vs. placebo | **5** |
| **8** | **Supplementary Fig 8:** Leave one out meta-analysis of GA at delivery <37 weeks after exclusion of Alizadeh et al, 2022, dydrogesterone vs. placebo | **6** |
| **9** | **Supplementary Fig 9:** Leave one out meta-analysis of cesarean section after exclusion of Keshtamandi et al, 2023, dydrogesterone vs. placebo | **6** |
| **10** | **Supplementary Fig 10:** Leave one out meta-analysis of Apgar score <7 at 1-minute weeks after exclusion of Keshtamandi et al, 2023, dydrogesterone vs. placebo | **7** |
| **11** | **Supplementary Fig 11:** Trial sequential analysis (TSA) of the gestational age at delivery. | **7** |

**Table S1**. Details of the search strategy

| Databases | Restrictions | Term | Number of items found |
| --- | --- | --- | --- |
| PubMed | Title and abstract | #1= (“6-Dehydro-9 beta-10 alpha-progesterone” OR “6 Dehydro 9 beta 10 alpha progesterone” OR “Dehydrogesterone” OR “Isopregnenone” OR “Duphaston” OR “dydrogest*” OR “oral progesterone” OR Didrogesterona OR Didrogesterone OR Dydrogesterona OR Dydrogestérone OR Dydrogesterone OR Dydrogesteronum OR Gestatron OR Hydrogesterone OR Hydrogestrone OR Isopregnenone OR “oral Pregnenedione”)  #2= (preterm OR premature) AND (delivery OR birth)  #3= #1 AND #2 | 60 |
| Scopus | Title, abstract and keywords |  | 225 |
| WOS | Topic |  | 80 |
| Cochrane | Title and abstract |  | 53 |
| Total | |  | 418 |


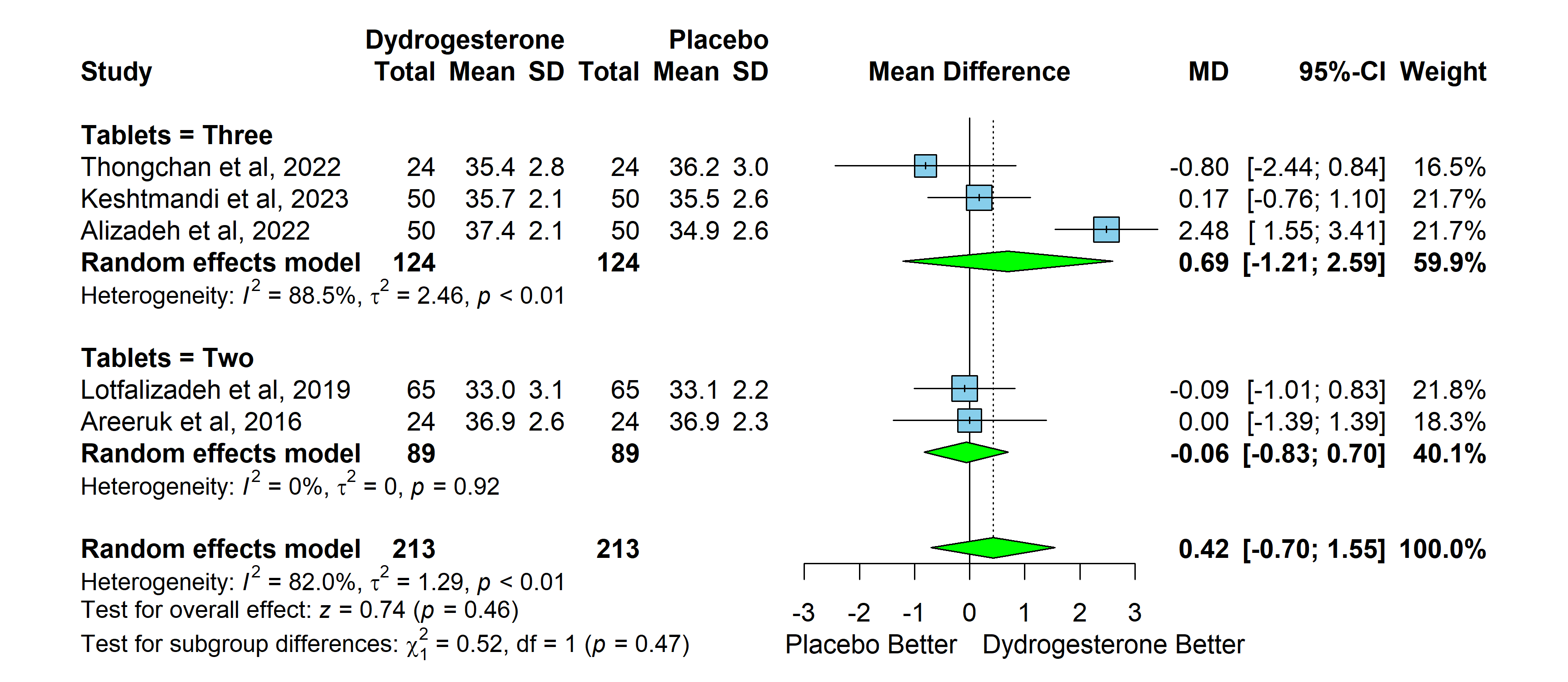


Supplementary Fig 1: Subgroup Analysis by Tablets for gestational age at delivery (Three Tablets vs. Two Tablets).


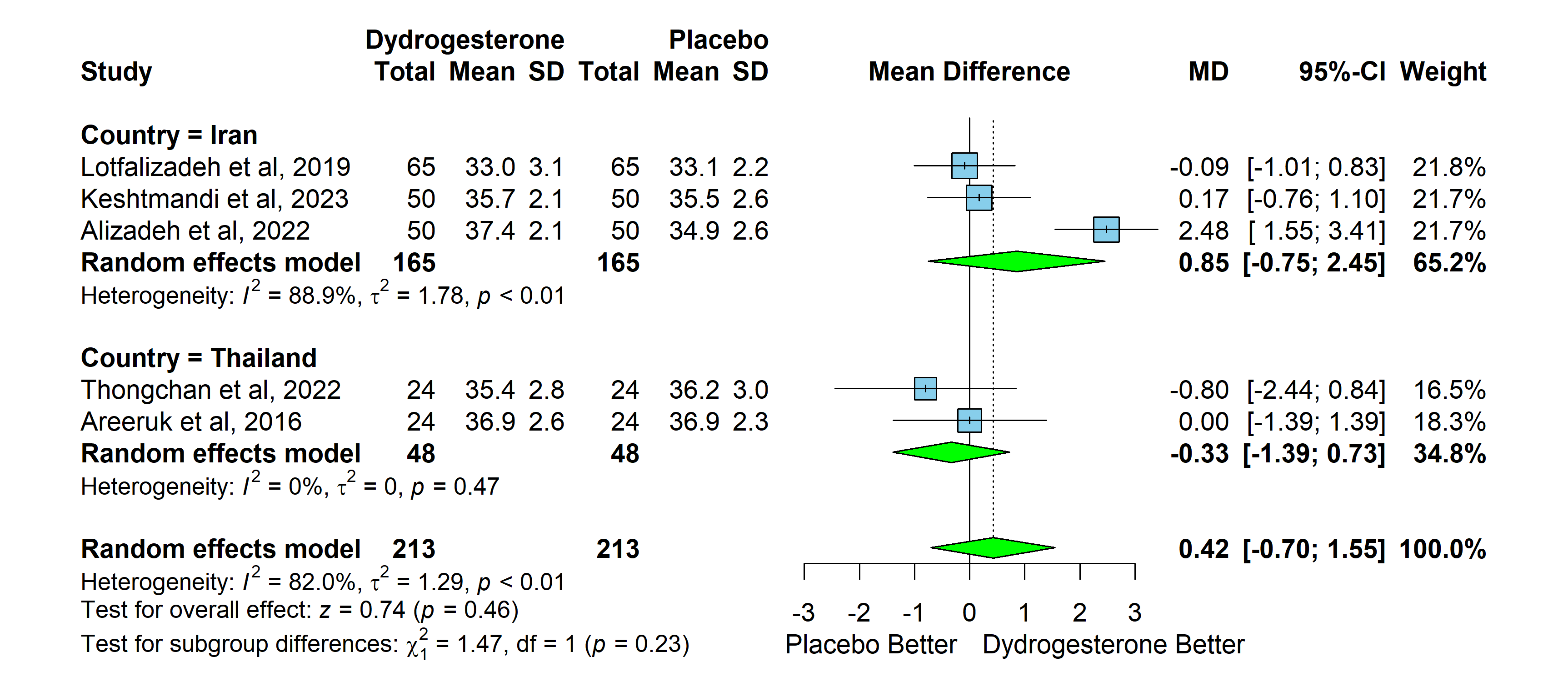


Supplementary Fig 2: Subgroup Analysis by Country for gestational age at delivery (Thailand vs. Iran).


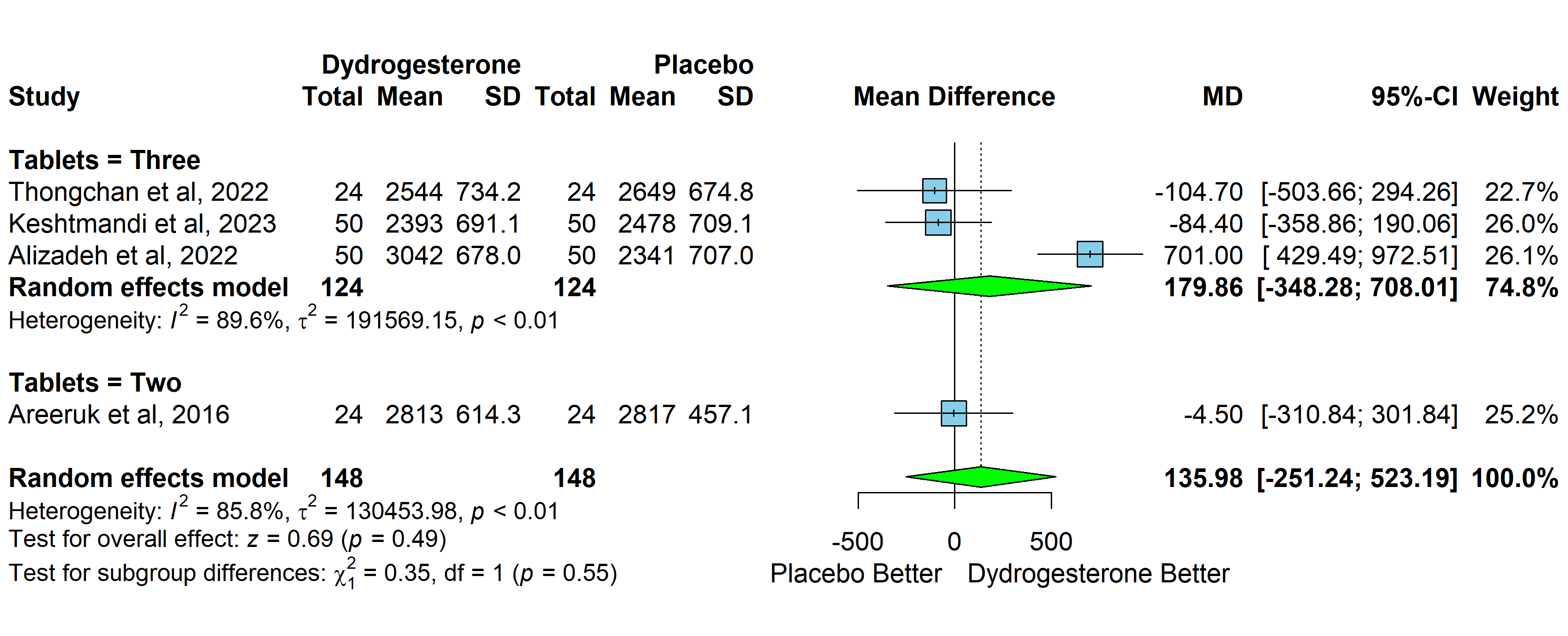


Supplementary Fig 3: Subgroup Analysis by Tablets for Birth Weight in grams (Three Tablets vs. Two Tablets).


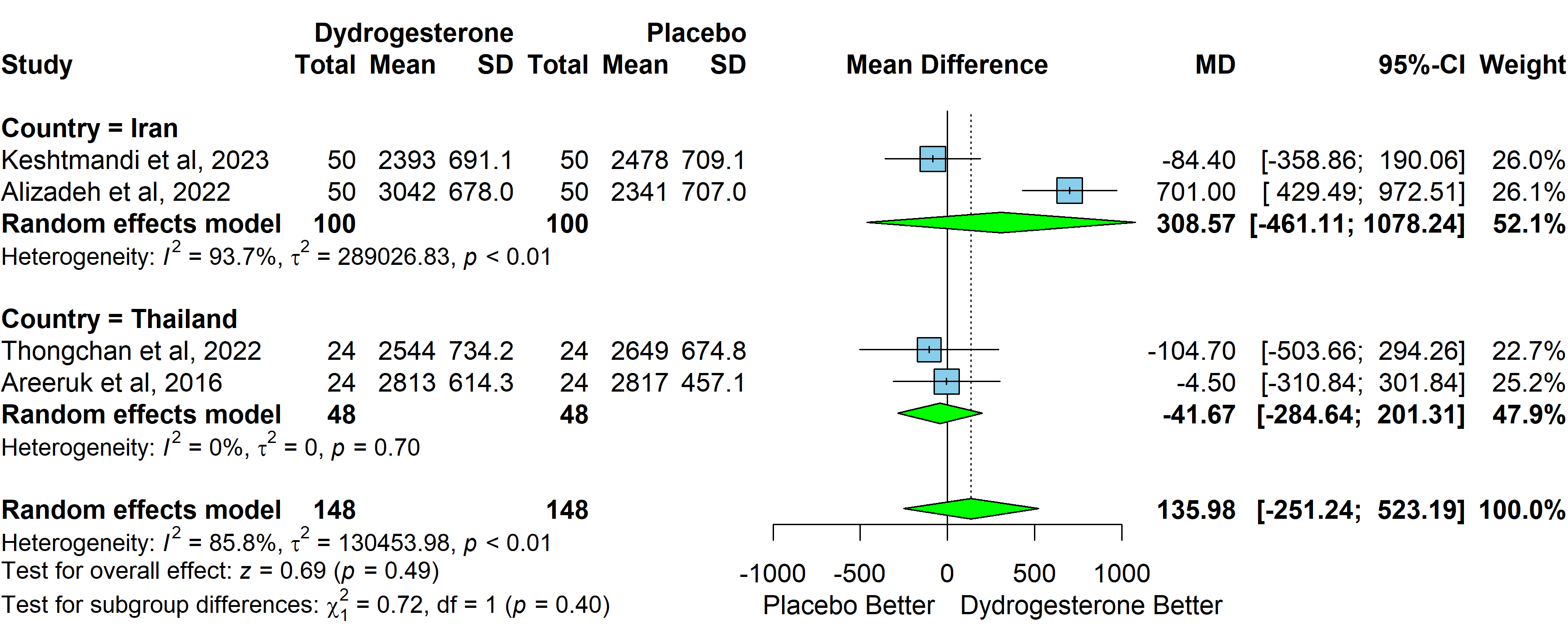


Supplementary Fig 4: Subgroup Analysis by Country for Birth Weight in grams (Thailand vs. Iran).


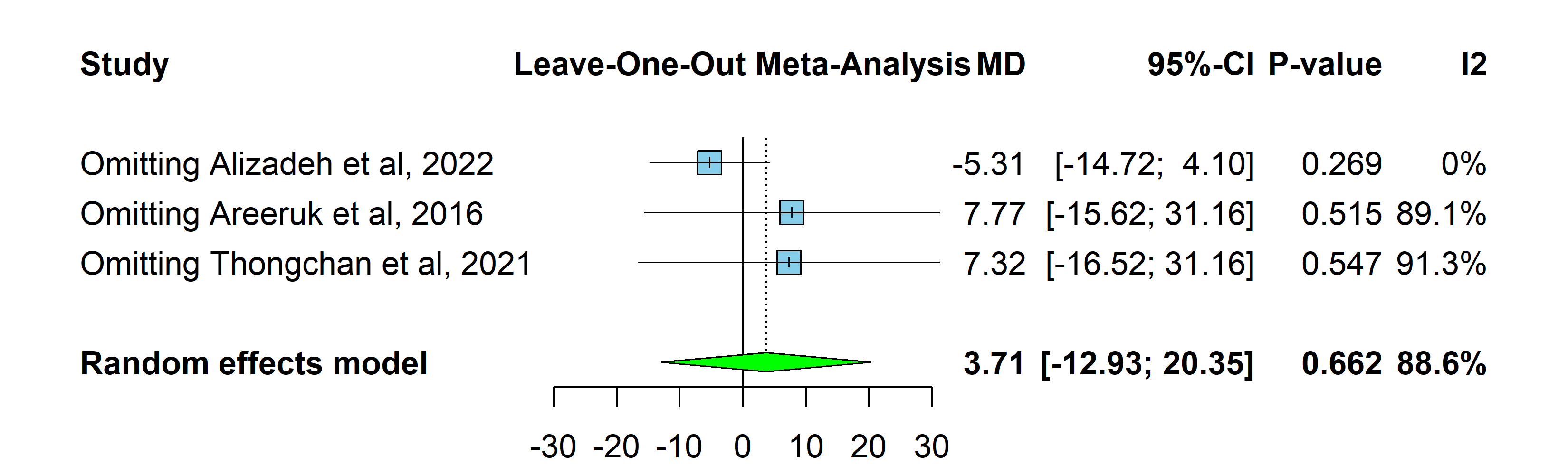


Supplementary Fig 5: Leave one out meta-analysis of latency period after exclusion of Alizadeh et al, 2022, dydrogesterone vs. placebo


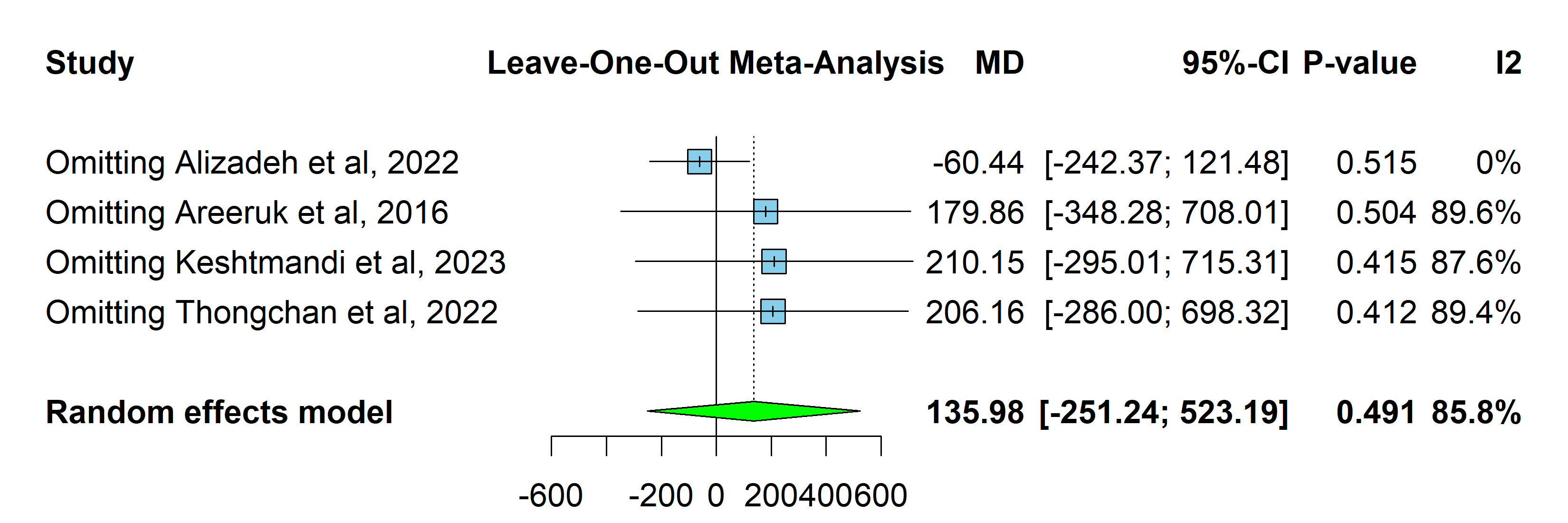


Supplementary Fig 6: Leave one out meta-analysis of birth weight in grams after exclusion of Alizadeh et al, 2022, dydrogesterone vs. placebo


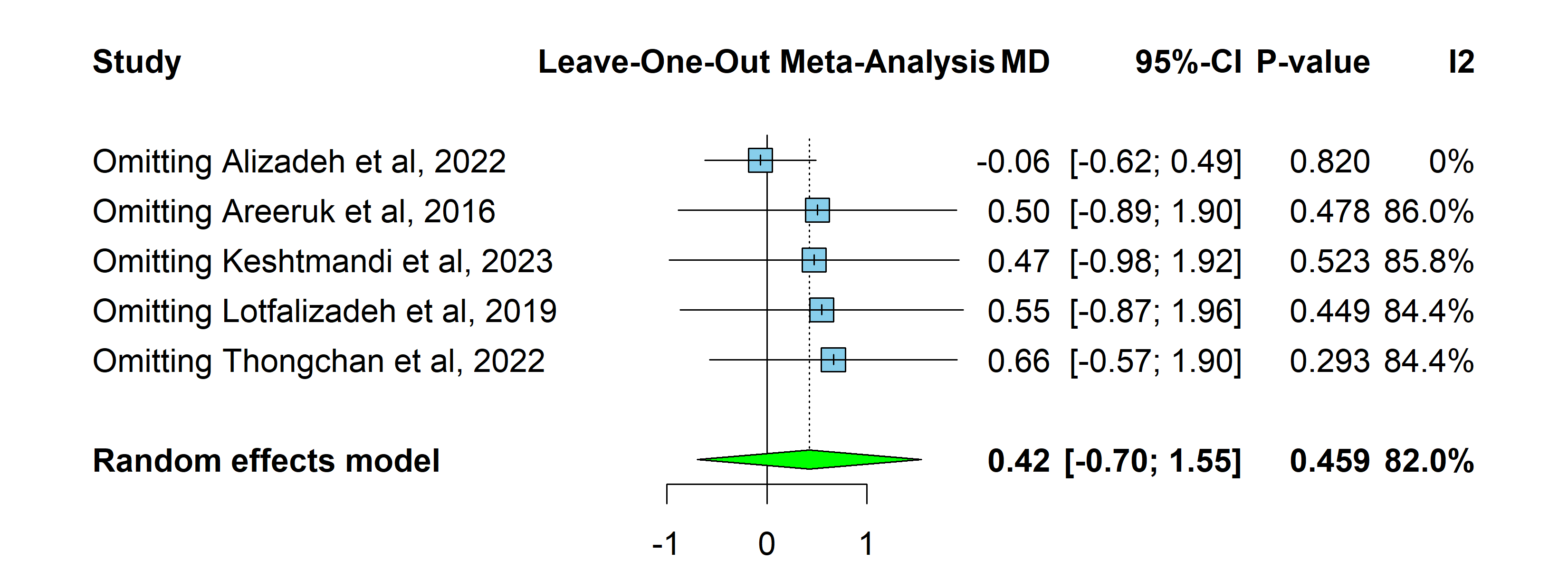


Supplementary Fig 7: Leave one out meta-analysis of GA at delivery in weeks after exclusion of Alizadeh et al, 2022, dydrogesterone vs. placebo


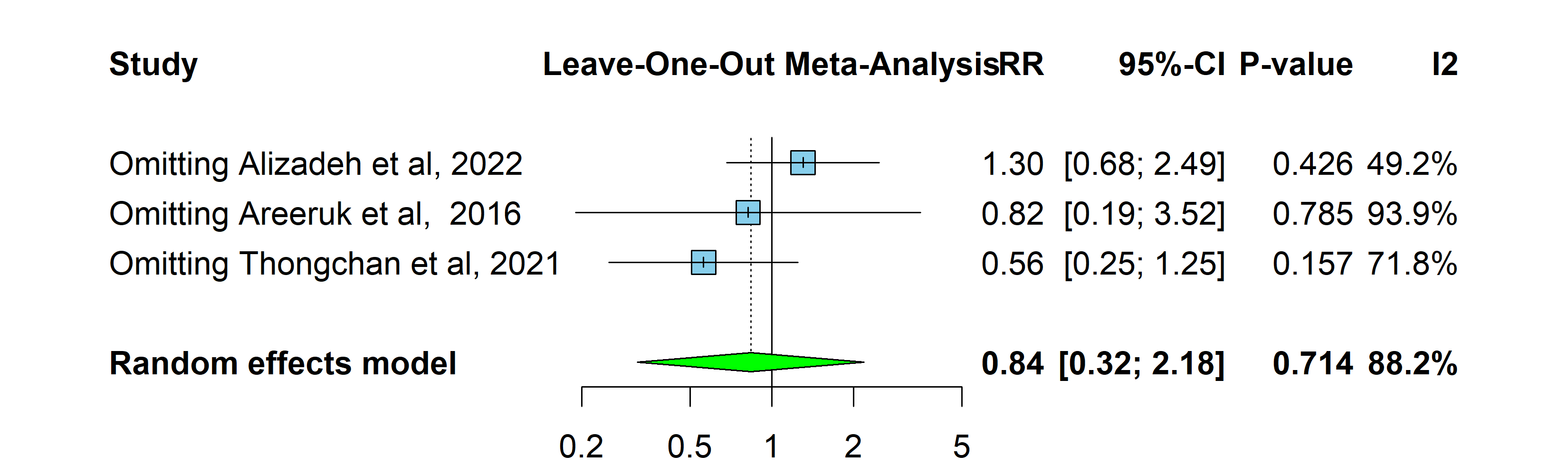


Supplementary Fig 8: Leave one out meta-analysis of GA at delivery <37 weeks after exclusion of Alizadeh et al, 2022, dydrogesterone vs. placebo

*
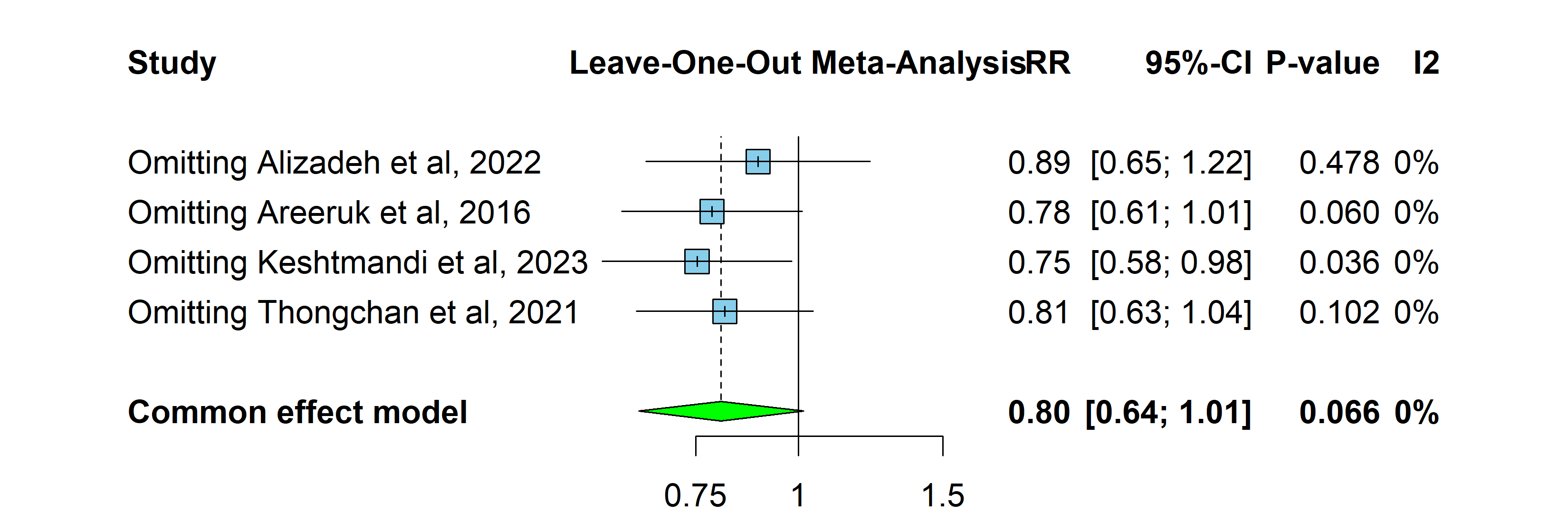
*

Supplementary Fig 9: Leave one out meta-analysis of cesarean section after exclusion of Keshtamandi et al, 2023, dydrogesterone vs. placebo


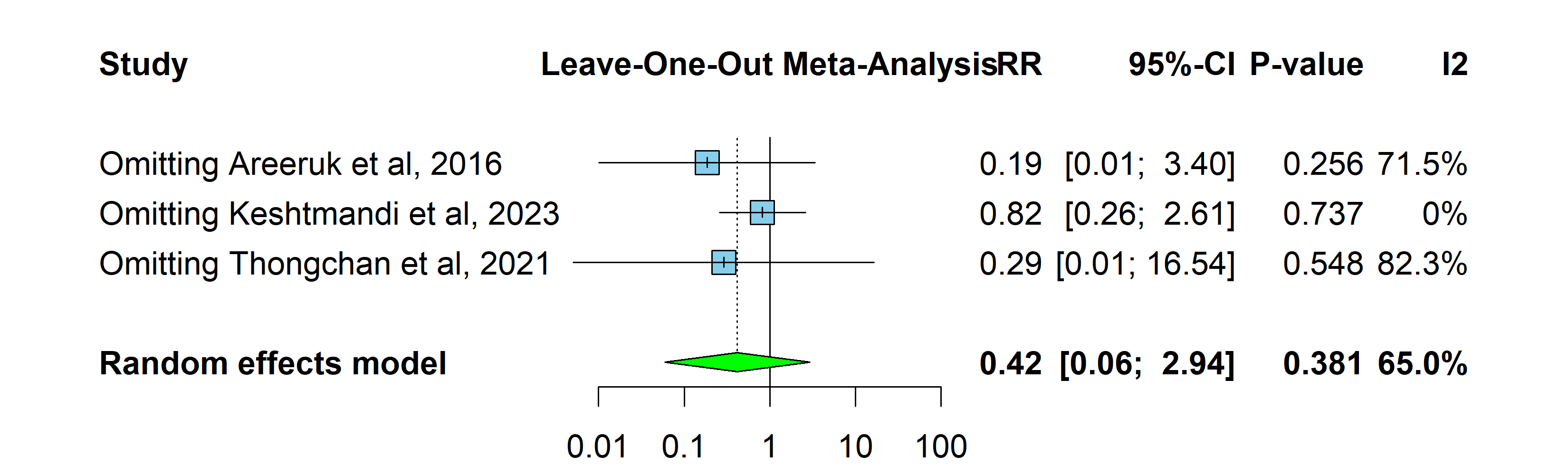


Supplementary Fig 10: Leave one out meta-analysis of Apgar score <7 at 1-minute weeks after exclusion of Keshtamandi et al, 2023, dydrogesterone vs. placebo


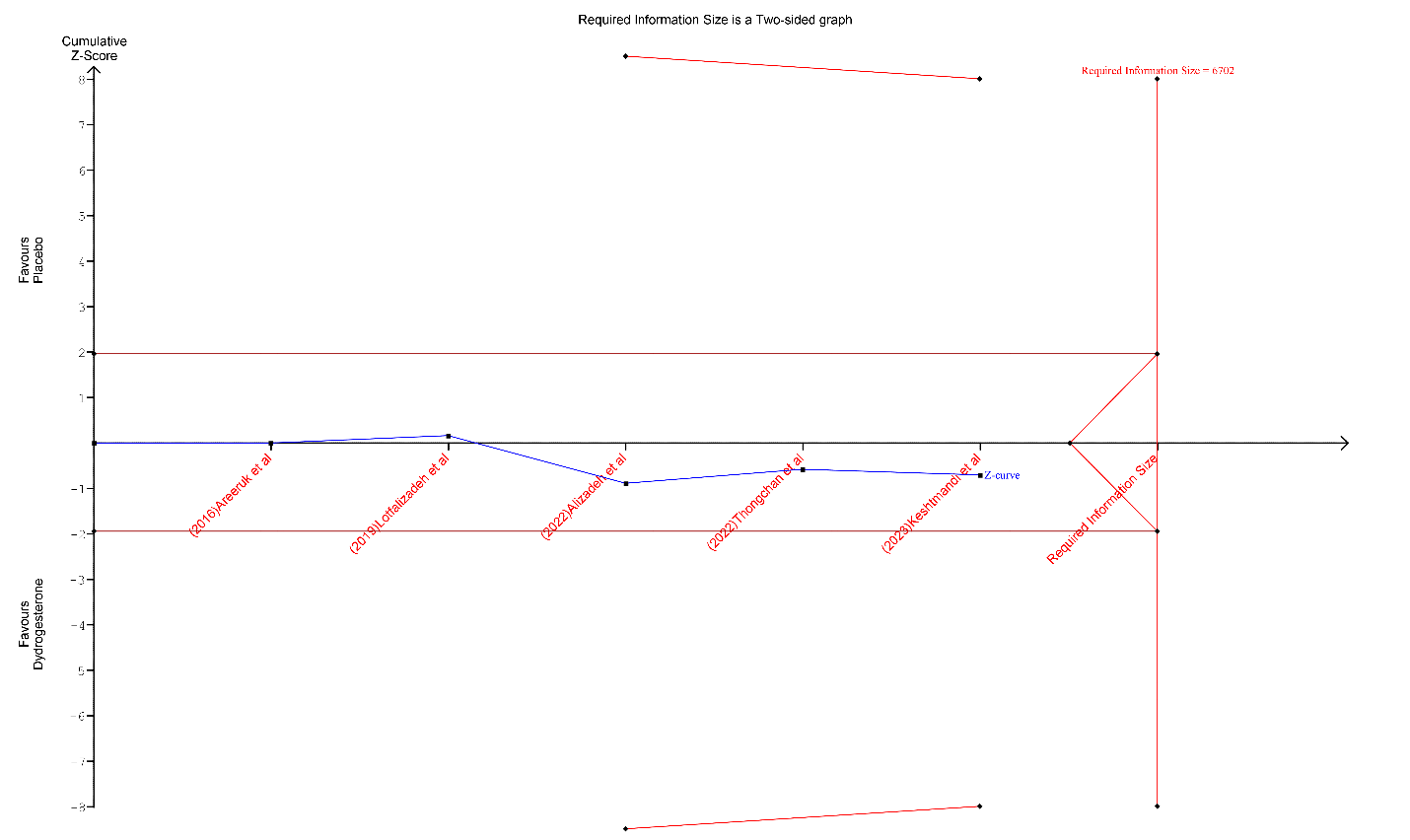


Supplementary Fig 11: Trial sequential analysis (TSA) of the gestational age at delivery.
